# Obituary

**Published:** 1912-03-16

**Authors:** 


### Obituary.

The death is announced of Sir Francis Cruise, at the age of seventy-seven. The deceased physician attained to most of the chief honours which the Irish profession offers to its heads. A quarter of a century ago Sir Francis was President of the Royal College of Physicians in Ireland, and in 1896 he received the honour of knighthood. He was also Physician in Ordinary to the late King Edward VII., and was decorated by the present Pope. Sir Francis Cruise was of a type of Dublin physician familiar in the pages of Lever, but now almost extinct; that is to say he was not only an eminent physician, but a cultured scholar and a thorough man of the world. He was one of the first to write authoritatively on balneology, and his first-hand acquaintance with European health-resorts was acquired at a time when few physicians had taken the trouble to pay personal attention to this branch of therapeutics. In particular the fame of Contrexéville is due very largely to his influence and recommendation. His non-professional interests were great and his general culture very extensive. It suffices to mention his devotion to music, of which art he was an expert connoisseur and devotee. As an antiquary, Sir F. Cruise will be long remembered for his researches into the authorship of the *Imitatio Christi*. Nowadays, secure on his foundation of patient research, it is difficult to realise that this question was one of great doubt and difficulty. The establishing of the fame of Thomas à Kempis was with him, says the *Times*, the great passion of his life; and it is of interest that at Kempen, near Düsseldorf, the grateful inhabitants have named a street after the deceased physician.

An interesting career has just been closed by the death of Inspector-General E. E. Mahon, C.B., R.N., at the age of sixty. After his professional studies at St. Mary's Hospital he entered the Navy in 1878, and was on service in the Zulu war of the following year. Then he again landed in a Naval Brigade, and fought against the Boers at Laing's Nek and Majuba; for gallantry in the latter disastrous action he was recommended for the Victoria Cross, and was specially promoted. Then he went with the Marines to the Egyptian War of 1882, and was present at all the principal battles of that campaign. Yet once more he served with a Naval Brigade, this time in Burmah in 1887, and was present at the capture of Mandalay. Unfortunately he contracted beri beri at a later stage of his career; and although he recovered, was obliged to give up the Naval Service.

